# Cultural “Blind Spots,” Social Influence and the Welfare of Working Donkeys in Brick Kilns in Northern India

**DOI:** 10.3389/fvets.2020.00214

**Published:** 2020-04-29

**Authors:** Tamlin L. Watson, Laura M. Kubasiewicz, Natasha Chamberlain, Caroline Nye, Zoe Raw, Faith A. Burden

**Affiliations:** ^1^The Donkey Sanctuary, Devon, United Kingdom; ^2^Centre for Rural Policy Research, University of Exeter, Exeter, United Kingdom

**Keywords:** working equids, brick kilns, welfare, blindspots, donkeys, culture

## Abstract

Non-governmental organizations (NGOs) work across the globe to improve the welfare of working equids. Despite decades of veterinary and other interventions, welfare issues persist with equids working in brick kilns. Engagement with all stakeholders is integral to creating abiding improvements to working equid welfare as interventions based purely on reactive measures fail to provide sustainable solutions. Equid owners, particularly those in low to middle-income countries (LMICs), may have issues such as opportunity, capacity, gender or socio-economic status, overriding their ability to care well for their own equids. These “blind spots” are frequently overlooked when organizations develop intervention programs to improve welfare. This study aims to highlight the lives of the poorest members of Indian society, and will focus on working donkeys specifically as they were the only species of working equids present in the kilns visited. We discuss culture, status, religion, and social influences, including insights into the complexities of cultural “blind spots” which complicate efforts by NGOs to improve working donkey welfare when the influence of different cultural and societal pressures are not recognized or acknowledged. Employing a mixed-methods approach, we used the Equid Assessment Research and Scoping (EARS) tool, a questionnaire based equid welfare assessment tool, to assess the welfare of working donkeys in brick kilns in Northern India. In addition, using livelihoods surveys and semi-structured interviews, we established owner demographics, socioeconomic status, ethnicity, religion and their personal accounts of their working lives and relationships to their donkeys. During transcript analysis six themes emerged: caste, ethnicity, inherited knowledge; social status, and impacts of ethnic group and caste; social status and gender; migration and shared suffering; shared suffering, compassion; religious belief, species hierarchy. The lives led by these, marginalized communities of low status are driven by poverty, exposing them to exploitation, lack of community cohesion, and community conflicts through migratory, transient employment. This vulnerability influences the care and welfare of their working donkeys, laying bare the inextricable link between human and animal welfare. Cultural and social perspectives, though sometimes overlooked, are crucial to programs to improve welfare, where community engagement and participation are integral to their success.

## Introduction

Working equids play a key role in the livelihoods of many inhabitants in LMICs. There are ~55 million mules and donkeys globally (1)^1^ and India still relies on over 500,000 donkeys and mules (2)^2^ to provide draft power to support the livelihoods of parts of India's rural and urban population. Donkeys and mules have been associated with poverty and low status for many hundreds of years (3) and are commonly the draft animals of choice for people working in brick kilns. Working donkeys in brick kilns have had many decades of resource provision, though long-term sustainable improvements to working donkey welfare has been largely unsuccessful (4) and policy makers rarely recognize their socio-economic and cultural value (5–7).

With a rapidly expanding human population requiring homes and other building, brick kilns are an integral part of India, being a visible industry in India's rural and peri-urban environments, where 125,000 kilns (8)^3^ provide 10% of global brick production (9). Brick production involves numerous processes starting with molders who fashion the clay into brick shapes. These “green” bricks dry in the sun until being stacked and moved to the kilns for firing. Once fired and cooled, bricks are removed from the kiln and stacked ready for distribution. Each stage has specific workers involved in the process starting with molders, then firemen and stackers. Stackers move the bricks, both unfired and fired, across the kilns using working equids. Traditionally donkeys convey bricks on the sites, being nimble and small enough to negotiate the narrow entrances of the brick kiln itself. In some areas, where sites allow, access by larger mules or tractors is increasing productivity and efficiency, though the areas accessed for this study were still utilizing mainly donkeys. An estimated 10–23 million people in India work in brick kilns as bonded laborers^4^, 40% are women and 20% are children under 14 years old (11). The brick kiln industry completely ignores the input of women and children, whose presence goes unrecorded, their earnings paid directly to their husbands (11, 12). Bonded laborers are commonly low-caste, illiterate and poor, whilst kiln owners are generally high caste, literate and wealthy (11).

India has a built-in system of inequality, known as the caste system, developed around 3,500 years ago and associated with Hinduism^5^ (14), and which leads to social stratification (8). The system uses ideologically driven criteria to rank people's location on this hierarchy. Rights to ownership of land, business, and access to education gets progressively reduced the further down the hierarchy one descends. Unequal access to land, education, lack of social and occupational mobility, exclusion from access to resources, and restricted human and civil rights, means that those in lower castes and Scheduled Tribes^6^ will remain in this position (16, 17).

This study examines the complexities of the lives of the poorest in India, exploring the Hindu caste and Scheduled Tribe systems, and how cultural “blind spots” create challenges for NGOs attempting to target donkey welfare when the influence of different cultural norms are not recognized or acknowledged. Here we explore the links between human culture and working donkey welfare, in line with the concept of “one welfare.” “One welfare” emphasizes appreciation of context and other contributing factors which influence animal welfare, and how linkages between animal welfare, human well-being and the environment should guide creation of holistic approaches to welfare interventions (18, 19). Delving into perceptions of status within these systems, we examine whether inherited knowledge, believed to be inherent in certain ethnic groups, influences the welfare of donkeys working in brick kilns. We evaluate the implications of status on the lives of donkey owners, putting particular emphasis on women. Investigating the contribution of migratory employment, a common denominator of unskilled brick kiln workers, toward feelings of shared suffering between owners and their animals and whether feelings of compassion of owners toward their donkeys links with better donkey welfare. Finally, we evaluate the role of religion, species hierarchy and the effect this may have relating to working donkey welfare in brick kilns of northern India.

## Materials and Methods

### Study Site and Demographics

This study was conducted between 30 April and 14 May 2018, in Gujarat state, on the Western coast in Northern India. The 14 brick kilns included in this study were in the region surrounding the city of Ahmedabad, which has a human population in excess of 60 million (20) Most workers we saw were family units, otherwise known as “Jodi” labor, including women and children (age range of under 10 years old to late teenagers). The large number of brick kilns in this region guided the choice of area for the study and the presence of a local partner [Donkey Sanctuary India, (DSI)] which enabled access to kilns, logistical support and interpretation services.

### Data Collection

#### Quantitative Data

A mixture of both qualitative and quantitative methods were employed for this study. Livelihood surveys (LSs) (Appendix 1 in Supplementary Material) were conducted to gather data on demographics, accommodation, income, household composition, and ethnicity of donkey owners working within the kilns. Only donkeys were present in the kilns surveyed. Each survey was recorded digitally using Open Data Kit (ODK) collect (21); data were uploaded to the server when connected to the internet. Data collection occurred when most owners had finished work (8 a.m.−1 p.m.). Most workers within the kilns work throughout the night to avoid high daytime temperatures. Prior to administering the survey, the interviewers ensured those participating had been able to carry out any essential household tasks (e.g., people were being fed, animals had finished work and were being fed and watered before release for grazing).

To assess the welfare of donkeys in the brick kilns, we used the Equid Assessment, Research and Scoping (EARS) tool, which is a comprehensive, questionnaire-based method of assessment built upon the numerous scientific equid welfare assessment tools available (22). EARS assessments were conducted on approximately half of the donkeys belonging to each owner. Completion of EARS assessments for, on average, six animals was equivalent to completion time for each semi-structured interview (SSI) before donkeys were taken away for grazing, so logistics, particularly time constraints, guided this decision. Data were collected on body condition score (BCS) and general health (GH) (Appendix 2 in Supplementary Material) were used. BCS is a good indicator of health and welfare (23) and “general health” gives broadest overview of the condition of the animal. Although subjective, BCS is reliable and repeatable (24, 25). BCS was quantified using a scoring system developed specifically for donkeys [see Appendix 2 in Supplementary Material; (26)], where 1 = poor (very thin), 2 = moderate (thin), 3 = ideal, 4 = overweight (fat), 5 = obese (very fat). GH scores were assigned using the EARS tool. Animals were assigned to a category of “good,” “fair” or “poor,” with a visual assessment of the presence and location of wounds, signs of harmful practice (e.g., signs of tethering or hobbling) and presence of injury [full details of the GH assessment can be found in Appendix 2 in Supplementary Material]. A trained assessor performed welfare assessments whilst SSIs were taking place. Data were inputted into the ODK Collect app (21) on a tablet or phone and transferred to a database once the equipment reached an internet connection.

Quantitative data were uploaded to the software package R version 3.6.1 (27) through R Studio Version 1.2.5019 (28). Descriptive statistics were extracted and graphs plotted using tidyverse, which enables packages to work effectively together for data manipulation and visualization (29). Tidyverse includes the packages used in this study: tidyr (30); readr (31); dplyr (32); forcats (33); and ggplot (34). Our sample size is small but representative of the nature of this study, due to the difficulty encountered in accessing working donkey owners who were able to offer their time to participate. Consequently, we did not perform statistical analysis as we do not want to over-interpret results, and have relied on exploratory analysis and visual inspection of the data to provide a preliminary indication of any association between owners and their donkeys' GH and BCS. Although this means we are not able to draw absolute conclusions, our results give an indication which could be more deeply explored using larger datasets.

To ensure that differing sample sizes did not bias our results regarding the association between welfare and both ethnic group and compassionate language, we used bootstrap analysis, a non-parametric technique which employs random resampling with replacement (35). Data were tested using 1,000 replicates of three random samples per owner. Terms incorporated each possible combination of ethnic group or compassion (“compassionate” or “not compassionate”), and BCS or GH. One term for BCS was removed for test assessing both compassion and ethnic group as it only contained one donkey (BCS = 4). All terms except for one (Compassionate; BCS = “very thin/poor”) fell within 95% quantiles of the bootstrap dataset, supporting a lack of sampling bias in our dataset for all other terms.

#### Qualitative Data

One to one SSIs (see Appendix 1 in Supplementary Material) were conducted to allow for richer data capture of the personal experiences of workers. Each participant was allocated a unique code to ensure all data were anonymized. Interviews were conducted in Hindi by either LMK and TLW, using Indian interpreters from DSI; they were recorded by dictaphone and translated into English during the recordings. Qualitative data were transcribed, uploaded and analyzed by TLW using Nvivo (Nvivo 12 qualitative data analysis software, V.12.5.0, QSR International). Initially a deductive approach to coding was considered; religion became a prominent discussion point during interviews and as such its link to welfare felt worthy of further exploration. As interviews were coded however, open coding was adopted as it gave scope to capture more in-depth narratives. The iterative inductive approach allowed analysis and identification of emerging themes based on the qualitative data, saturation was reached when no new codes were being generated. A co-author (LMK) reviewed themes for agreement on findings and outcomes, any differences between coding were discussed and agreement reached before themes were considered finalized.

We recognized that owners used different language in the context of “compassion,” so following completion of fieldwork, we categorized owners as “compassionate” or “not compassionate.” To do this, we analyzed the language owners used toward their donkeys within interviews. “Compassionate” owners recognized their donkeys were suffering with them in the kilns, understood their donkeys felt pain, owners vocalized they shared pain and suffering, spoke of their donkeys as if part of their human family, that they felt pity for their donkeys, recognized each donkey's individuality, and spoke about their affection and bonds to their donkeys. Those classed as not compassionate spoke in utilitarian terms, about using donkeys as tools and did not express affectionate language about their donkeys. Donkey welfare was compared between the “compassionate” and “not compassionate” groups.

### Ethical Approval

Research was carried out under the research policy and guidelines of The Donkey Sanctuary and received approval from the executive team therein. Recruitment of participants was on a voluntary basis, due to illiteracy of participants verbal informed consent was gained from each person and recorded via dictaphone. All participants were anonymized and were given the right to withdraw within 2 weeks of interviews being carried out. To withdraw participants were informed to contact DSI, no participants exercised this right. Non-invasive techniques were used to assess donkey welfare.

## Results and Discussion

### Livelihood Surveys

LSs were completed for 37 donkey owners, 28 of which completed semi-structured interviews. Welfare assessments were completed for 219 donkeys (stallions = 189, mares = 30) from 33 donkey owners (mean = six donkeys assessed per owner). SSIs, livelihoods surveys and welfare assessments were typically undertaken simultaneously by TLW and LMK. On some occasions, owners became unavailable due to tiredness, or they changed their mind and decided not to be interviewed which made it impossible to collect both the LS and SSI for every single owner. All owners used their own donkeys to carry bricks by pannier in the brick kilns. Brick kiln work was the primary source of income for 97% of participants. An overview of owner demographics can be found in Table 1.

**Table 1:** Demographic information of donkey owners in the study.

**Demographic**		**Percentage**
Gender	Female	14
	Male	86
Ethnic Group	Vanjara	78
	Salaat	22
Ages	18–30	27
	31–50	49
	Over 50	24
Household composition (Adults)	Two	32
	Three	11
	Four or more	57
Household composition (children)	One	6
	Two	24
	Three	27
	Four	16
	Five	16
	Six and over	11

The majority of donkey owners were Vanjara (*n* = 78%), fewer belonged to the Salaat ethnic groups (3% consisted of the Waadi sub-group of Salaat) (*n* = 22%). No other ethnic groups owned donkeys, though other ethnic groups worked in distinct sections of the kilns molding bricks, stacking and firing bricks when made (Alvi, Ansari, Mansuri Muslims). Kiln managers (Prajapati and Sakya Hindu) and kiln owners (Prajapati Hindu) were all higher caste.

Household composition showed all households surveyed contained at least two adults and at least one child. All children had to remain with their families within the brick kiln for the duration of the season (typically 6–8 months). Outside of kiln season, three donkey owners lived in rented accommodation outside of the kiln season (8%), with all others owning their own home (92%), very few (*n* = 4; 11%) donkey owners owned land. All donkey owners lived on site at the kilns during the kiln season.

Only 23 (62%) donkey owners gave the distance they had traveled to work in the kiln, 39% traveled over 100 km (one owner 1,000 km), 26% traveled 50–100 km, and 35% traveled under 50 km to the kiln. This work removed all workers from their familiar communities due to economic necessity.

### EARS Welfare Assessments

The 33 participants owned on average eight donkeys with 11% owning six and under, 38% owning 7–9 donkeys, 51% owning at least ten donkeys. We conducted welfare assessments for at least half of each owner's group of donkeys; animals assessed were selected at random.

Donkeys showing moderate/thin BCS (BCS = 2) formed the largest cohort of 45, and 27% were “very thin/poor” (BCS = 1). A quarter of all the donkeys showed “ideal” BCS (BCS = 3), and only one donkey was categorized as “fat” (BCS = 4).

The largest cohort of 57% showed fair GH, 24% had poor GH and 19% had good GH.

All donkeys showed signs of harmful practices^7^ related to the use of tethers or hobbles to restrict movements when not required for work.

Of the 33 owners for which both livelihoods surveys and welfare assessments for their donkeys were available, 25 were Vanjara and eight were Salaat (including the three Waadi). There were 174 and 45 donkeys welfare assessed, respectively for Vanjara and Salaat owners.

**Figure 1:**
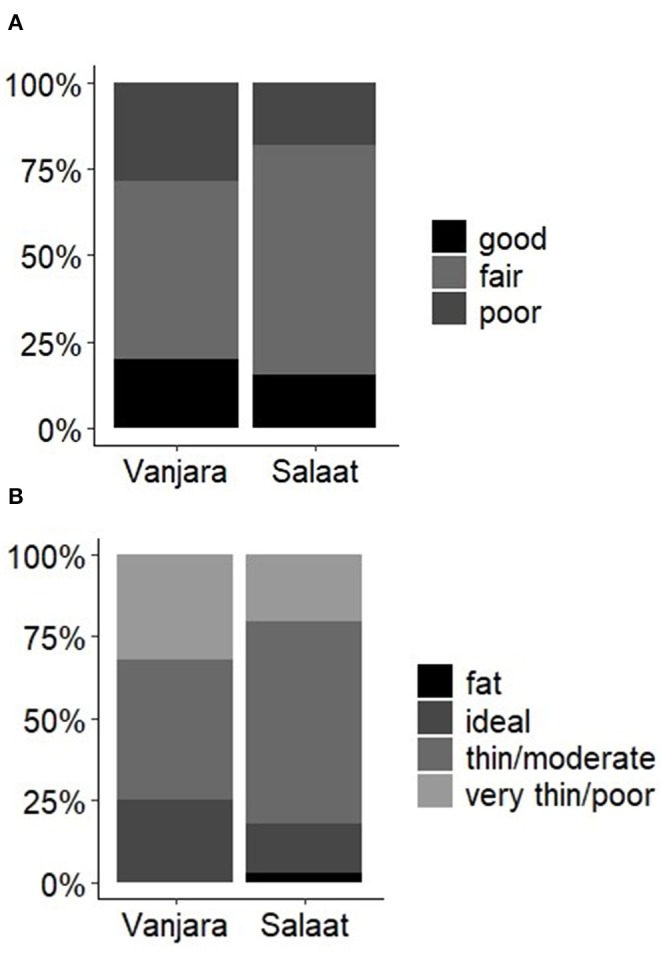
Percentage of donkeys that obtained **(A)** “good,” “fair,” and “poor” general health scores and **(B)** each body condition score, grouped by the ethnic group of the donkey owner.

Overall, both groups of donkeys had individuals that showed poor GH and low BCS (Figures 1A,B). In a comparison of GH and BCS between donkeys owned by different ethnic groups, the Salaat community overall had more donkeys with a fair general health score than Vanjara, but proportionally fewer with good scores (Figure 1A). Salaat also had fewer donkeys showing “very thin/poor” body and “ideal” condition scores than the Vanjara (Figure 1B).

**Figure 2:**
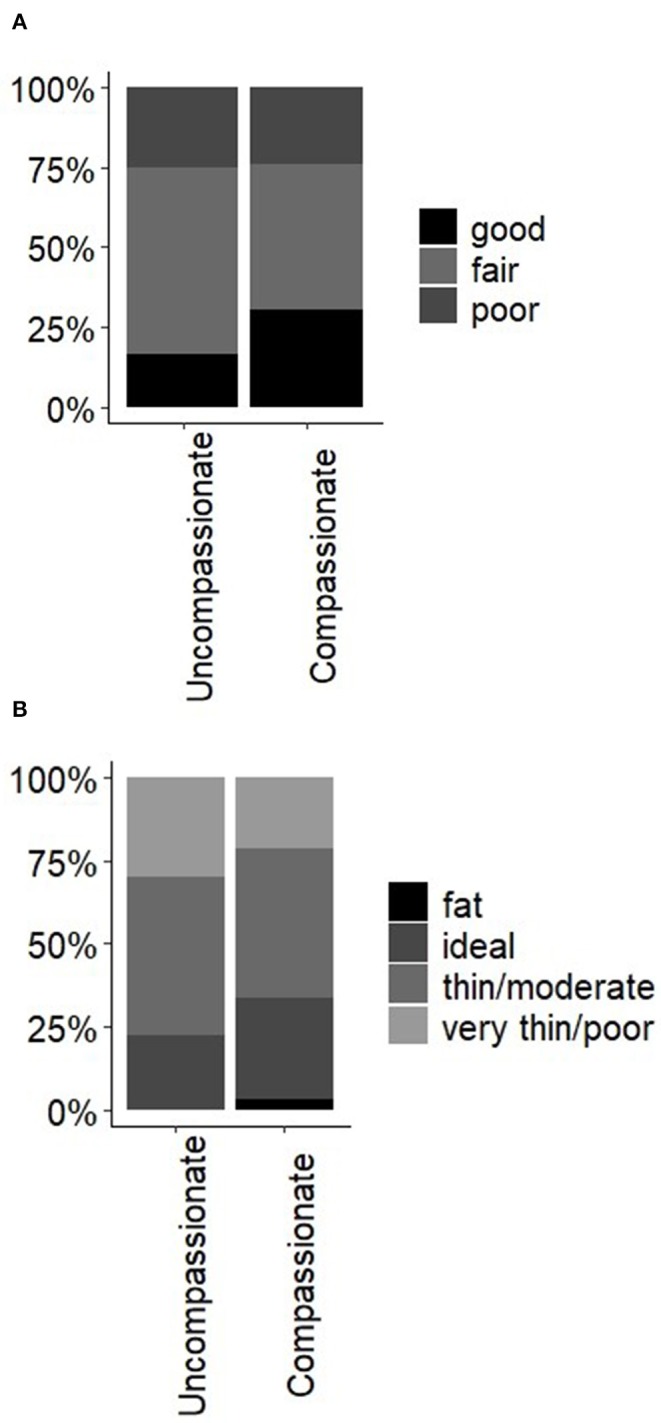
Percentage of donkeys that obtained **(A)** “good,” “fair,” and “poor” general health scores and **(B)** each body condition score, grouped by whether owner used or lacked compassionate language when relating to their donkeys.

Thirty three percent of owners spoke using language indicating compassion or affection toward their donkeys (listed as “compassionate” in Figures 2A,B). Those speaking compassionately, owned donkeys with slightly better welfare compared to those owners lacking compassionate language. In the compassionate category, one donkey was classed as “fat” (BCS = 4), but 43% were in the “ideal” category and 14% “thin/poor” (Figure 2A). Owners that spoke with compassion also had more donkeys in the “fair and good” categories for GH (Figure 2B).

When using the bootstrap method, one term (BCS = very thin/poor) fell outside the 95% quantiles. There were 7 donkeys within this category, 57% (*n* = 4) of these belonged to one owner, potentially biasing results for this particular category. As this owner used compassionate language, if sampling had been more evenly spread (i.e., fewer samples from this owner), we may have observed a stronger positive association between better body condition and the use of compassionate language.

### Qualitative Data

Six key themes emerged when analyzing transcripts: caste, ethnicity and inherited knowledge; social status, ethnic group and caste; social status and gender; migration and shared suffering; shared suffering, compassion and donkey welfare; religious belief, species hierarchy and donkey welfare. Each theme and their relevance to donkey welfare are discussed in detail in each individual section.

#### Caste, Ethnicity, and Inherited Knowledge

The Salaat group tended to keep mares and foals as they felt they were easier to handle, and to travel with, though one group of owners at one kiln kept only stallions. As with other equids, donkeys are called foals if they are under 1 year old. There were obvious signs that donkeys under 2 years old were working, as were heavily pregnant mares despite owners declaring they did not work any donkeys in these categories as noted in field notes:

8th May 2018 MS Kiln
I asked the owner which donkeys he was currently working, he pointed to them all and said all were working apart from the young foal, a pregnant mare, and two foals in the following group owned by his other family member. During assessment, I found one young donkey (1–2 years old) had been working, contrary to the owner's assertion, and two very heavily pregnant mares showed signs of work despite the owner claiming one had not been working for 2–3 months. There were signs of harness sweat marks on all donkeys, apart from the very young foals. These foals were extremely difficult to assess, head shy, and received rough, aversive handling from owners.

Vanjara kept only stallions, their handling varied from “aggressive” to “light and confident” within EARS criteria. They were also working young animals, in the 1–3 years old category, though from assessments all were over 2 years old. Though donkeys appear mature at 2 years old, their bones are only fully mature at 3–4 years old and should only bear weight and endure hard work when they are 5–6 years old to avoid permanent damage (36).

The Salaat, an ethnic group classed as low caste, are known for being farmers using bullocks; their donkey knowledge was reported anecdotally by DSI as being better than other ethnic groups. This anecdotal reporting could misdirect focus toward the more socially favored class the Salaat, which could perpetuate stigma and guide resources toward or away from different groups.

Although they have a low status within Indian society, low caste people, such as Salaat, are at least considered a part of society and suffer less discrimination compared to those from tribal communities who are left socially distanced and isolated (17, 37). The Vanjara, (commonly referred to as Banjara), are nomadic people who fall within the Scheduled Tribes classification. During the colonial era, Vanjara were designated a criminal caste which still negatively influences perceptions of them in present day India (38, 39). The Vanjara suffered systematic persecution mainly because the tribe maintained independence from the rest of society, were highly mobile within the country and had no set orientation with regards to religion making them difficult to control, and as such were subject to much distrust (40). State level “civilization” reduced the status of these people, women particularly, through the integration of nomadic tribes to Indian society (37, 41). Vanjara traditionally used bullocks for transporting goods, but they also used camels, donkeys, horses and mules (42) so potentially have substantial inherited knowledge of animal care for various species including donkeys.

When speaking to owners about inherited knowledge regarding donkey care, some Salaat owners did not seem to have family histories of ownership but their communities were able to offer some support:

“We go with families only not to work, we learn from others, they teach us how to tie the animals […] I learn from others whatever they are doing we learn.”

This Salaat owner described how he had to learn through mistakes about their care:

“Yes but what to do sir, the donkey hit me here, he kicked me on my head […] but I still learned sir but now they're fine, once I got a kick on my head.”

A lack of inherited knowledge may well have given this owner the freedom to experiment without pressures from family expectations. For example, he built a holding pen to confine his donkeys, the only owner throughout the field study to do so. This management practice gave donkeys slightly more freedom of movement by negating the requirement for tethering or hobbling. When asked why he had designed the pen:

“Because there are 6 donkeys inside when they fight or do something or when they fall down its leg will get entangled, he may break; if he becomes lame then we will have to work with less donkeys.”

Social stratification leaves poorer inhabitants in communities further segregated from mainstream society, irrespective of the country they inhabit, by creating barriers to information (43). This limited access to resources, particularly educational, may encourage a community or familial self-reliance (44) where inherited knowledge is relied upon to guide procedures, in this instance donkey husbandry. This path of knowledge transfer perpetuates knowledge, beliefs or practices irrespective of their basis in fact (45, 46), which has obvious implications for donkey husbandry when owners use practices which may or may not be good for donkey welfare.

#### Social Status—Impacts of Ethnic Group and Caste

When asked specifically about how donkeys impact perceived status, all apart from three Vanjara owners felt having donkeys increased their social status, all apart from one Salaat owner were either unsure or felt it did not have an effect. This is not the whole picture though.

One owner mentioned that they thought those people who were only slightly richer seemed to look down on donkey owners: “[…] so if people have tractors, JCB's^8^ they think […] they are slightly higher than the donkey owner.”

Many owners felt that donkeys increased their social status but aspired to stop owning donkeys and own machinery instead:

“Only when [I] become rich can [I] afford to have tractors and JCB.”

Therefore, despite there being some increase to perceived status, in reality owners realized their work was hard and paid poorly:

“Because I am illiterate, I am supposed to do the donkey work, if I would have studied, I would have done [another] job”

They expressed concerns that donkey ownership becomes negative when discussing their children's marriage potential. It seems most disadvantageous when discussing the marriage of sons, as they fear most other families do not want to give their daughters to donkey owning families:

“The girl's families may reject the boys.”

This is obviously a common fear; a female participant voiced her plan to overcome this issue:

“[.] You know when someone asks you for a girl from somebody else's family that time they say we are not going to give the girl to you because you own donkeys […]so I would hire a tractor from somebody else [.] and let them give the girl to me.”

Some families also do not want to give their daughters to donkey owning families, the reasons did not stem from status, but from concerns about the workload:

“I will only give my daughter only to Salaat who doesn't have donkey […] Oh my daughter will work to die, she will always have to work and she will die working, working in the Salaat family if she works with the donkey.”

The owners seemed to link the owning of donkeys less with the status of the animals themselves but more profoundly with feelings of suffering, poverty, and hard work they felt trapped within due to the Indian hierarchical caste-based economy and bonded labor typical of the brick kilns. During interviews, the subject of status becomes complicated, it may not link to ethnic group but more due to how these economically marginalized people feel about the manner in which they are perceived or wish to be perceived. Years of living at the tough end of the caste system may heighten human perceptions about unfair prejudice and bias through their personal experience, history and reality (47).

#### Social Status and Gender

Women are of lower status compared to men, according to India's patriarchal society, influencing their perceived roles. Women are expected to take care of the home, childcare, water, food, and work, and here she is not viewed as an individual in her own right being perceived of inferior status (48). During this study, male owners informed us, and anecdotally we were informed that women did not have time to take care of donkeys, but are expected to take care of the home, children, and work:

“Amrit [husband] does all the feeding, grooming, and taking care of the donkeys; she does all of the household chores.”

We observed women on occasion feeding, watering and handling the donkeys so this does not necessarily complete the whole picture. Women spoke of being unable to find time to look after donkeys during the brick kiln season but having to look after them during the off-season when their husbands have to go off to labor elsewhere:

“She doesn't find time in the brick kilns […] because here she has to fetch water from a faraway place, in the village they have water in their home, and [in the brick kiln] she has to bring fuel for the fire […]they have to go to the jungles to collect [.]She takes the donkeys for grazing when she goes home. During the off-season when her husband, Amrit, goes out for some work, then she is the one who takes the donkeys for grazing and takes care of the donkeys.”

Women commented that they did not feel confident looking after donkeys out of the kiln season when their husbands needed to find other laboring work:

“If he goes for any labor work, she stays at home and she is not able to manage those donkeys.”

An ability to care for donkeys when returning to their own villages has other welfare concerns driven by a lack of suitably trained and experienced vets:

“When they are in the village when the donkey gets sick and they call the doctor, the one who treats the buffaloes and cows, they are not able to treat the donkey”

“the doctor used to come and treat us very nice but he transferred, he left so nowadays we have to use the other people […]they may be government vets or private vets that come to see the buffalo but he says there was one good doctor in Dahor until he got transferred.”

In addition, a lack of empowerment or funds to ring a vet if they do have one:

“She waits for her husband to come home, because he's the one who can make call to the doctor […] She says that she would like to do but she doesn't have a mobile with her, so she has to wait for her husband to come home so that she can tell that the donkeys sick and we have to call the doctor.”

When given the opportunity women seemed keen to be involved:

8th May 2018 MS Kiln
During one visit, a donkey had its teeth rasped as it had a visible pouch on the side of its face. The vet there called the male owner over to look as he was treating the donkey and offered the man a chance to put his hand in its mouth. I had to ask that the female owner be given the same opportunity; she was thrilled to do so and ran up to me afterwards saying she did not realize donkeys have dental problems too.

None of the women had received any training or guidance to look after donkeys despite their obvious involvement in caring for them. This may be due to social bias where India's patriarchal society gives men predominant access to resources (48, 49), or from the assumption that women appear too busy to have time to be involved. De Mello (8) articulates that systems of exploitation usually linked to other forms of discrimination, so where there is a class or caste system there may be racism, homophobia or oppression of women. Lower caste women and those within Scheduled Tribes take position at the bottom of caste, class and gender hierarchies. Women in Vanjara caste (when they were long-distance traders) used to be considered of much higher status, they were involved with all economic, social and cultural activities and had influence over decision making including those around animal care (41). This is no longer the case in Indian society.

The under-representation of women within this study (see Table 1) supports the findings of Mudege et al. (50) and Valette (6), where the common perception of men as household heads and women as illiterate helpers within the household, reproduces this gender bias, denying women access to information and training, and making women less willing to share their voice. Social influence seems to direct resources most commonly to men as they are considered the primary carers for the donkeys. Perhaps less frequently noticed caring for donkeys within the kilns or perhaps due to India's patriarchal system, the women remain largely invisible. Presupposition that women will marry and care for households can leave them at greater risk of illiteracy than men, which sustains the assumption that women may struggle to understand information given. Training resources traditionally offered to men leave women in a challenging predicament if left to care for donkeys alone, particularly when veterinary professional capacities are limited in many rural areas. In this study, women were in direct daily contact with their working donkeys, and expressed a lack of confidence in being the sole carers outside the kiln season. Lack of inclusion, low status and invisibility of women has implications for working donkey welfare, particularly when workers return to their home communities where women may be relied upon to take over donkey management with limited access to adequate veterinary support. If interventions by organizations only target men, donkey welfare may be put at risk as women unquestionably contribute to animal husbandry (6).

#### Migration and Shared Suffering

All donkeys observed within the kilns showed signs of harmful practices, with open wounds and scarring related to the use of hobbles and tethering. These practices ensure donkeys remain within the confines of the area where they are required for work, but it is also indicative of the challenging nature of the situation. Owners spoke about needing to keep the donkeys from roaming onto other neighboring lands; donkeys doing so were subject to violent attacks from free-roaming dogs:

“[.]It gave birth to a normal foal—after two or three days the foal would have run near the wild dogs—we lost a three-day-old foal.”

Alternatively, their donkeys and owners found themselves vulnerable to attack from neighboring landowners:

“[.]if it goes to some green fodder place the people hit our donkeys, so I have to restrain them, so he is saying the people beat our donkeys, so if they go to the field.”

“[.]Sometimes those people whack them also you know when their donkeys enter somebody else's field, that's ok with the donkeys but they come and beat these people.”

This conflict may be purely to protect crops from donkey foraging or from community tensions where the transient nature of workers and their animals leads to distrust or open hostility.

The danger to workers, particularly women, within these communities we described in field notes. Female vulnerability when using exposed locations to perform daily tasks and personal hygiene is well-known, despite this women use these spaces to socialize with other women and gain respite from their homes (51). The women in this study may be particularly at-risk being migrants in an unfamiliar, potentially hostile environment, so although being able to spend time with other women, the socialization is more of a necessity to maintain safety than a reward:

10th May 2018 AB Kiln
Water is 12 km away from site, as the kiln owner will not provide it. The women have to walk together to get water as it is not safe for them to do so alone.

According to owners, donkeys have more freedom at their home location, though it is unknown if this is the case:

“We don't tie them [during off season]—only, only in the brick kiln, the whole day roam.”

“[.]They're always grazing, off season they're always out.”

As only two owners owned any land, we can at best surmise that any improved situation for the donkeys when they return home is due to their familiarity and acceptance within their home communities. However, without observing the management of donkeys in their home environment it is impossible to know whether in practice this difference in management occurs.

Lower castes and Scheduled Tribes are forced to endure migratory, transitory work where poor pay and instability affects family lives. This work keeps children poorly educated and continues the cycle for subsequent generations. Social and land constraints of India's caste system limit their incomes, being unable to diversify employment or living on agriculturally poor land (if they own any at all); (16). Illiteracy and poverty leave workers vulnerable to exploitation, bonded and child labor are common themes throughout the brick kiln industry, where no workers' rights, limited checks, and low societal status means they are largely invisible to the rest of Indian society (12). Families have to leave the security of their own communities where they are commonly struggling with food and monetary poverty because of a lack of land or through living on marginal land, which is unproductive (52). Brick kiln owners using the services of “jamadurs” (labor agents) frequently scout out those in this precarious situation to be employed in brick kilns (12). The absence of support when people have to migrate outside their communities increases ease of exploitation by the industry, their illiteracy, invisibility to the state and lack of community cohesion whilst working away from home leaves the workers, particularly women, vulnerable (11).

#### Shared Suffering and Compassion

Some owners clearly remembered happier times, referring to their traditional way of life before the kiln work became essential due to lack of employment in their historical trade:

“We were in our home making them [stone carving/ grinding], our children were all going to school, we are all settled life, but here in this job we're always moving. We are away from our house we are working for eight hours, eight months and that, so in that business we had a good life, our children could go… but now in this life we are always like moving.”

This discontentment in their present lives showed when some owners spoke about their donkeys, about their shared suffering:

“He's saying working with, we are feeling bad because we are, the donkeys are also suffering with us now for the work.”

Although there were fewer donkeys in the poor BCS category with owners who spoke with compassion there were still 14%, so despite owners feeling sympathy for their donkeys:

“She […] feels pity that they are speechless animals and that they can't speak, and they can't express their feelings, but she has that sympathy and empathy both for the animal.”

They understood a lack of money was driving their own and their donkeys' suffering, and they would give them more food and shelter if they had the capability to do so:

“[.] If I have more money one day I will build a house for my donkey, I'll put fans, he is saying if I have my money I will build a house that's why they all are laughing and then he said I'll put fans for them.”

“He is saying I will feed them very well […] and also, I can – I will not work them all times, I will feed them if I have more money.”

Owners felt shared suffering:

“He is saying we just like my child, in pain, I feel the same.”

Owners attempted to maintain their donkeys' health through difficult circumstances, owners' health statuses were also poor through malnourishment, injuries and pain; something noted in field notes:

2^nd^ May 2018 BS Kiln
When pointing out a donkey's visible wounds to an owner the owner raised his bare hands showing them to be raw and abraded [from handling thousands of bricks per day without any protection], he looked thin and tired, his clothing dirty and worn. It is difficult to know where to start, human, and donkey welfare here is awful.

The donkey populations within the kilns did include many animals with poor welfare, most had either injury, wounds, and/or poor nutrition and all were subject to the harmful practices of tethering and hobbling. The migratory nature of these communities means organizations cannot work consistently with them, and the lack of community cohesion limits peer support and effectiveness of interventions. This in addition to the lack of ability for decision-making due to cultural constraints, lack of autonomy within their working environment and limited access to resources within the kilns make improvements difficult to achieve. Donkey welfare runs concurrently alongside the shared suffering of their human counterparts, only focusing on donkeys leads to an unsustainable outcome for both.

Behind every working animal are owners and families whose lives are restricted by poverty, low status and restricted access to resources. De Mello (8) asserts that human and animal suffering and exploitation are different sides of the same coin. Those systems limiting human economic and social mobility, such as the caste system, also work to oppress and restrict animals. Where humans within the brick kiln industry face subjugation and exploitation to give other humans profit, animals working with these people are subject to the same violation. Porcher (53) noted that scientific considerations on animal welfare fail to take into account the suffering felt between animals and carers in some agricultural systems depending on how intensive it is, and the degree of autonomy farmers and workers are given. This sense of shared suffering could easily extend to workers and working donkeys in brick kilns, where work is hard, debilitating and lacking in any sense of autonomy. The importance of comprehending the emotional significance of “sentient commodities” or livestock to their carers has been identified (54, 55). This affects the dynamic relationship between them via the subjective experiences throughout their animals' life span and is of great importance when trying to improve welfare.

The shared suffering of owners and their donkeys shows how vulnerable both populations are to exploitation. Many owners traveled over 100 km for work, leaving the security of familiar communities, facing risk of attack from neighboring communities should their donkeys become loose, and leaving women vulnerable when trying to access basic resources such as food and water. Owners discussed their happy memories of previous work before the kilns, their feelings of empathy sharing suffering with their donkeys who also had to work hard in the kiln. Empathic language and kindness seemed to have a small but positive association with better donkey welfare in this study.

People in dire circumstances, such as those discussed throughout this study, will struggle to make sustained improvements to donkey welfare. Vulnerability of both to exploitation, of being away from familiar support and at risk from a lack of community cohesion and sometimes direct hostility toward affected humans and donkeys alike. Human-focused NGOs were absent from kilns we visited, but there have been targeted interventions in other areas of India where migrant workers are given valuable guidance to access governmental support, and empower community driven groups successfully targeting people before being exploited by brick kiln owners (11). Collaboration with other agencies to empower workers and improve their access to support and resources would be a viable option for consideration when looking for long-term donkey welfare improvement. In addition, building owners' capacity and opportunity may in turn influence owner motivation to undertake interventions themselves by changing behavior, increasing feelings of empowerment, capability and development of appropriate beliefs both individually and within the community (56). These approaches would work in contrast to direct provision of expert services, and are most likely to have the most sustainable impact, though this has to be undertaken in a culturally sensitive manner, appreciating the complex problems these marginalized communities face (57).

#### Religious Belief and Species Hierarchy

We asked owners from both ethnic groups about a necklace many owners wore around their necks. This necklace depicts a deity worshiped by multiple faiths, known as Shitala Mata, or Sitala (
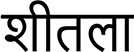
 ś*italā*), meaning “the one who cools” (58). This goddess rides a single donkey, or sometimes is enthroned and flanked by two donkeys. There is a belief she is able to cure some illnesses, historically smallpox before eradication. She protects owners, their families and their animals from harm:

“Even if we get any chicken pox or something we pray to this god, and the god will cure us, so it is very strong in our religion, donkey and this god, this is all part of our worship.”

This reverence seemed only acknowledged during particular rituals, rather than reflected throughout their daily care:

“It's a religious symbol for us, then we do the ceremony for the Shitala Mata, the goddess, then we do ceremonies for our donkeys also […] so on that religious festival time, we feed donkeys very good food, we decorate our donkeys.”

“During Holi festival, they are putting color, and during Diwali festival we make red, we put ghee, clarified butter, jiggery, we make laddus^9^ out of it, and then we give them on our religious days.”

The three owners quoted above, despite speaking and acting with reverence for their donkeys, still had animals with welfare concerns. Of their assessed donkeys, 80% had apathetic noted as their demeanor; 67% were BCS very thin/poor the rest being thin/moderate; 87% had eye and nasal discharge and unhealthy coats; all had badly fitted harness and signs of beating.

Owners obviously valued their donkeys' work despite their donkeys'welfare being poor:

“[.] Because of the donkeys, only we are eating everyday bread, so we will obviously. We really value them, because we eat our bread because of them.”

Owners may struggle to give donkeys the care they need due to lack of resources and knowledge, when welfare issues become too severe, these concerns are exacerbated by owners' aversion to euthanasia as described in field notes:

1st May 2018 AM Kiln
One donkey had been injured months before our visit. With a large part of the hoof absent the pedal bone was clearly visible when the hoof was raised. The owners hoped the hoof would grow down and stop the donkey being lame and “miserable” as he was their favorite donkey, Poppit. They said he had to be persuaded to work now by offering bread and would not do so without it. On the mention of euthanazia, the owners said they could not allow it; however, they did give permission for him to be removed to the DSI farm where he was euthanized later that day.

This has obvious implications for animal welfare; this owner was reluctant to have their donkey euthanized despite him struggling to walk (or work) due to the protrusion of the pedal bone through the sole of his foot. Despite obvious behavior changes, the donkey had a dull, withdrawn demeanor, where only food bribery would encourage him to walk; he was still required to work. The owners could not accept euthanasia despite overwhelming evidence that the donkey could not recover and would live only a life of pain until he finally died. It is unknown whether this is because of religious belief or economic need, or perhaps a combination of both.

Hindus believe all animals hold something of God so deserve not only respect but also reverence, all species should coexist within the same system where dominion and exploitation of other species is not accepted (59). This reverence, in practice, does not extend equally to all animals. In the Hindu caste system, non-human animals are also born into caste –some animals are revered, while others are stigmatized. Some cows are high caste like Brahmins; horses are warrior-castes like Kshatriyas, and dogs are low castes (60). Brahmin texts, even as far back as the third century, refer to donkeys quite negatively, “untouchables shall be outside the village and dogs and donkeys shall be their wealth” (47). Humans using donkeys for their specific trade, such as Kumhars (potters) in India, are low caste; with both human and donkey receiving equal ridicule and denigration (61).

Hindus reverence for the cow, specifically, when translated into their husbandry, does not necessarily show any visible signs of difference compared to the husbandry of other species (62). Native Indian breeds represent Hindu purity, while crossbred or Jersey cows suffer similar exploitation and abuse as humans marginalized in Indian society (63). Cows are restricted to marginal grazing where due to malnourishment they may struggle to reproduce (62), which creates a compromised immune system susceptible to heavy parasitic burdens and infectious disease (64). When animals are surplus to requirements and care withdrawn, the abandoned cattle may starve to death (62) or walked hundreds of miles to places where their slaughter is legal. This way, owners avoid direct links to their cow's demise and in doing so, maintain their own purity and ensure they do not interfere with the animal's own karmic journey (64). Fox (64) discusses the Hindu belief of “ahmisa” or non-violence and non-harming of animals, the basis of the sacred cow doctrine, which influences the health and welfare of cattle in India. These religious beliefs have a profound impact on other domestic animals, which, depending on the status of those animals such as unclean, or immoral, become life negating rather than life affirming (63, 65).

Hindu faith teaches that lives are on a continuum of life and rebirth. In each phase of life, individuals have to release themselves from Karma in order to progress to the next stage of their spiritual evolution. A life of devotion, kindness and love enables progression to the next level after death, so it is integral to an owner's karmic balance to ensure their animals are cared for well. Karmic balance is disrupted if life is cut short unnaturally, such as through euthanazia, as this is believed to hold back karmic progression (66). The lower castes and Scheduled Tribes follow religion and tradition closely (67); this may influence willingness to euthanize animals, though economic value will also likely have some bearing.

In this study, though purported to be subjects of religious reverence, donkey welfare was still sub-optimal. There are clearly contributing factors such as socio-economic pressures and lack of capacity, though it is probable that the authors have a westernized view of how religious reverence should manifest. In India animals are considered as a part of the whole, so although all life is revered, if you are in poor circumstances, animals are considered partners within the situation, having to struggle alongside. Religious reverence may not be enough to ensure good care, though it is difficult to assess this when the socio-economic situation of the owners hinders adequate care. Reluctance toward euthanazia may have some basis in religious belief, but people's socio-economic status must have some bearing, the fear of economic loss greater than acknowledging suffering.

#### Study Limitations

Our partner organization, DSI, enabled access to sites and provided interpretation during interviews. DSI had previously provided veterinary interventions to some of the kilns within the study, so we acknowledge that this may have influenced responses of some participants. Without this assistance, however, access to sites would have been unlikely, dangerous and extremely difficult.

Uneven sample size is due to the different representation of each ethnic group in this cohort, and variation in the number of donkeys owned. We accounted for this as far as possible when interpreting results.

## Conclusion

For too many decades equine welfare organizations worked as silos, focusing narrowly on the species within their own strategic remit with only a snapshot in time of the donkey's welfare status. Working with little or no comprehension of the life cycles of human or donkey actors is potentially missing the “why” welfare is being compromised, which could provide guidance for the “how” welfare could be improved. A lack of recognition of the pressures these brick workers face makes many interventions to improve welfare unsustainable and unrealistic. Involvement and collaboration with other agencies equipped to provide guidance and support for humans is crucial for improving the capacity of people to improve their own and their animals' circumstances for the long term, which can only serve to create a more solid foundation for ameliorating animal welfare.

There is a need to ensure that “blind spots” are acknowledged through a comprehensive cultural understanding of the people who receive support and interventions from NGOs. A systems-based, one welfare approach is urged to identify linkages and root causes of issues and support the development of sustainable collaborative solutions. Interventions should not rely solely on veterinary treatments or knowledge transfer interventions, but should be diverse and have the flexibility to respond to differing circumstances. They should include better comprehension of human behavior, human-donkey relationships and focus more on underlying causes rather than treatment of symptoms. Facilitating owners to develop their own solutions to agreed problems, would give people ownership and empowerment to succeed. That facilitation and support should include all actors relevant to working donkeys' life cycles is apparent, that it should extend to accommodate women is requisite.

This paper highlights the drawbacks of only focusing on one-half of an inextricably woven relationship, and of the difficulties sustaining long-term improvements due to the complex or “wicked” problems in working donkey welfare.

## Data Availability Statement

The datasets generated for this study will not be made publicly available. Data sets relating to EARS and livelihood surveys can be made available on request to the corresponding author. Full interview transcripts cannot as participants did not explicitly give consent for them to be made available.

## Ethics Statement

The studies involving human participants and animal study were reviewed and approved by The Donkey Sanctuary, Sidmouth, Devon, UK. Written informed consent was not provided because verbal consent was obtained and recorded, due to illiteracy of participants.

## Author Contributions

TW, LK, NC, ZR, and FB: conceptualization. TW, LK, and NC: methodology. TW and LK: investigation and data collection. TW, LK, and CN: data curation. ZR and FB: supervised the study. TW: data analysis and writing of original draft. LK: data analysis and revision of original draft. CN: reviewing original draft. All authors: manuscript revision and read and approved the submitted version.

## Conflict of Interest

The authors declare that the research was conducted in the absence of any commercial or financial relationships that could be construed as a potential conflict of interest.

## Footnotes

^1^FAOSTAT is the Food and Agriculture Organization of the United Nations. Data is listed as live animals under “asses” and “mules,” it is aggregated and may include official, semi-official, estimated or calculated data. For more information visit the FAOSTAT website http://www.fao.org/faostat/en/#data/QA

^2^DAHD is the Department of Animal Husbandry and Dairying, in the Indian Government's Ministry of Fisheries, Animal Husbandry and Dairying. Livestock censuses in India started in 1919, all data gathered by house-to-house collection for more information visit the DAHD website: http://dadf.gov.in/sites/default/filess/Basic%20Animal%20Husbandry%20and%20Fisheries%20Statistics%202017%20%28English%20version%29_5.pdf

^3^ASI (2017) Anti-Slavery International https://www.antislavery.org/wp-content/uploads/2017/09/Slavery-In-Indias-Brick-Kilns-The-Payment-System.pdf

^4^Bonded labor occurs when very low wages compel workers to approach employers for advances of income. This economic burden forces entire families to remain indebted to employers for years, the debt sometimes being passed on to each successive generation (10).

^5^India has numerous religions, but Hinduism has the greatest number of followers, 79.8% according to the most recent population census (13). https://www.census2011.co.in/religion.php

^6^Scheduled Tribes first appeared in the constitution of India in 1950. They form 8.6% of the Indian population (15). The nomenclature refers to a community holding “primitive traits, distinctive culture, geographical isolation, are backward and retain a lack of integration to the rest of Indian society”. For more information http://vikaspedia.in/social-welfare/scheduled-tribes-welfare/ministry-of-tribal-welfare

^7^Harmful practices can be due to physical harming from violent handling, beating or from common practices used to control the behavior or movement of animal – these differ depending on culture. In LMICs frequently use tethering or hobbling to restrict an animal's freedom to roam. Harmful practices may also be based around cultural beliefs where practices such as nostril-slitting and firing are incorrectly thought to improve an animal's ability to breathe (the former) or increase its strength (the latter).

^8^JCB stands for Joseph Cyril Bamford. J C Bamford excavators limited, universally recognized as JCB, is an English multinational corporation manufacturing equipment for construction, agriculture, waste handling and demolition. In this particular instance, they make reference to the JCB excavator.

^9^Laddus are an Indian sweet made from a mixture of flour, sugar, and shortening shaped into a ball.
