# Supplementary material for: Cultural “Blind Spots,” Social Influence and the Welfare of Working Donkeys in Brick Kilns in Northern India
Source: Front Vet Sci. 2020 Apr 29;7:214. doi: 10.3389/fvets.2020.00214 (PMC7201042; doi:10.3389/fvets.2020.00214)
Supplement: Supplementary file 1 [file Table_1.DOCX]

**Appendix 1.**

**1a. Livelihoods survey – survey designed for use in multiple projects so not all data used in this study.**

| **Name of the location** |  | | | | | | | |
| --- | --- | --- | --- | --- | --- | --- | --- | --- |
| **Date** |  | | **Time** | | |  | | |
| **Assessor name** |  | | **Participant ID** | | |  | | |
| **Do you consent to participating in this survey?** | Yes | No (stop the survey) | |  |  | |  |  |
| **How old are you?** | Under 18 (stop survey) | 18 to 30 | | 30 to 50 | Over 50 | | Over 18, prefer not to give specific age |  |
| **Gender** | Male | Female | |  |  | |  |  |
| **What is your current job role?** |  | | | | | | | |
| **Who is the head of your household?** | Me | Spouse | | Other (male) | Other (female) | | Unsure |  |
| **How many adults (everyone 18 and over) are in your household?** |  | | | | | | | |
| **How many male children (under 18) are in your household?** |  | | | | | | | |
| **How many female children (under 18) are in your household?** |  | | | | | | | |
| **What is your religion?** | Hindu | buddhist | | Muslim | Christian | | Jewish | Other (please specify) |
| **Do you own your own home, rent, or stay with family?** | Own | Rent | | Live with family or friends | Other (please specify) | | | |
| **Do you own or rent any other properties or land?** | Own | Rent | | Use land owned by family or friends | Do not own or rent | | Other (please specify) | |
| **Do you have any of the following in your household?** | Mobile phone | Radio | | TV | Computer/  laptop | | Motorbike | Car |
| **What is your education level?** | None | Read and write | | primary | secondary | | University/  college | Other (please specify) |
| **Are you currently taking part in / have plans to do further training? (Please specify)** |  |  | |  |  | |  |  |
| **If not, would you like to do any further training? Please specify)** |  |  | |  |  | |  |  |
| **Do the school-age children in your household go to school?** | Yes, all of them | Yes, some of them | | No |  | |  |  |
| **How often do they attend?** | Every day | Most days | | Occasionally | Other (please specify) | | | |
| **For the children that don't attend school, or only attend occasionally, why is that?** | Cannot afford fees | Cultural reasons | | Needed to help with household chores | Work with the mules | | Work in other income generation |  |
|  | Migrated away from school | School too far | | Education not necessary | Completed compulsory education | | Other (please specify) | |
| **Would you like your children to work with equines in the future or not?** | Yes | No | | Unsure | Other (please specify) | | | |
| **How long have you worked in your current role for?** |  | | | | | | | |
| **What did you do before your current role?** |  | | | | | | | |
| **What do you see yourself doing in 10 years time?** |  | | | | | | | |
| **How many days per week do work with equines?** |  | | | | | | | |
| **How many equines are you responsible for?** |  |  | |  |  | |  |  |
| **On average, how old are the equines you look after?** |  |  | |  |  | |  |  |
| **Are you looking after more or fewer equines than last year?** | More | The same | | Fewer |  | |  |  |
| **Why is that?** |  | | | | | | | |
| **Is your work with equines your main source of income?** | Yes | No | | Unsure | Other (Please specify) | |  |  |
| **If not, what is your main source of income?** |  | | | | | | | |
| **Do you have any other sources of income?** |  | | | | | | | |
| **How many years have you worked with equines for (in total)?** |  | | | | | | | |
| **Did your family work with / own equines when you were child?** | Yes | No | | Other (Please specify) | | | | |
| **Do you or your family own any equines now?** | Yes | No | | Other (Please specify) |  | |  | |
| **Who in your household owns/owned the equines?** | Me | Spouse | | Other male family member | Other female family member | | Other (please specify) | |
| **What do you do if one of the equines you own after is sick or injured?** |  | | | | | | | |
| **What do you do if one of the equines you look after is sick or injured?** |  | | | | | | | |
| **Who makes the decisions regarding care for the equines at work?** | Manager | Me | | Vet |  | |  | Other (please specify) |
| **Who makes the decisions regarding care for the equines at home?** | Me | Spouse | | Other male family member | Other female family member | | Other (please specify) |  |
| **Any general additional information?** |  | | | | | | | |

**1b. Semi-structured Interview questions – master sheet of questions used in multiple projects, some were not included in this study.**

**Equine owners**

Can you tell me about where you and your family are from?

How many of your family came here with you to the brick kiln?

How long have you worked in brick kilns?

Do all of the family members that came here with you work in the kiln too? What do they do?

How long have you worked with donkeys?

Why did you start working with donkeys?

How do you feel about working with donkeys?

How long have you owned the donkeys you have now?

How do you feel about the donkeys you own now?

What impact would it have on you and your family if you did not have a donkey?

How much of your family’s livelihood depends on your donkeys?

Do you think owning a donkey has an impact on a person’s social status/their status within their community? In what ways?

Who in your family is responsible for making decisions about the donkeys, and for looking after them every day?

What role do women in your family have in working with or looking after donkeys?

What will you do with them at the end of the season?

If you are choosing to sell some, how will you decide which ones to sell and which to keep?

What do you do with the money from selling your donkeys?

How do you feel when you sell one of your donkeys?

Where do you buy your donkeys?

What do you look for in the donkeys you buy (cost, size, sex, characteristics…)?

What impact does it have on you and your family if the monsoon season comes early and the kilns close early?

If you keep your donkeys, what happens to them in the monsoon season when the kilns are closed?

What was the process for you to be recruited to work in the brick kiln this season?

How are you paid for your work? In advance at the start of the season, or weekly/monthly per quota?

If you are paid in advance for your work, are you able to work that off by the end of the season? What happens if you can’t transport enough bricks to work it off?

How do you communicate with your donkey and get it to work for you?

Who do you go to for advice with or help for your donkey if it is sick or has a problem?

Do all your donkeys work every day?

What would the impact be on you if one of your donkeys died or got very sick and could not work anymore. What would you do?

Do your donkeys do any other work or are they used to help with household chores after the have finished in the kilns for the day? What sorts of things do they do?

What happens to your donkeys during their resting time? (are they tethered/hobbled/put in stables, or allowed some time to roam and graze)

How important is it to you to keep your donkeys healthy?

What sorts of things do you do to keep your donkey healthy?

Is there anything that stops you from giving your donkey what you think it needs to stay healthy?

We notice you have a religious symbol worn on a necklace around your neck showing a deity riding a donkey; does reverence of this deity influence what you feel about your donkeys?

Do you treat your donkeys differently to other animals because of this deity? If so how?

Who do you go to if you need help or advice on your donkey?

What do you know about Donkey Sanctuary India? Have you ever used any of their services? Which ones, and what was the outcome/what did you learn?

Are there any human development organisations that work in this kiln, and have done any projects here? Can you tell us about them?

What do you think is the future for donkeys in brick kilns?

**Brick kiln workers (non-equine owning)**

Can you tell me about where you and your family are from?

How many of your family came here with you to the brick kiln?

How long have you worked in brick kilns?

Do all of the family members that came here with you work in the kiln too? What do they do?

What impact does it have on you and your family if the monsoon season comes early and the kilns close early?

What was the process for you to be recruited to work in the brick kiln this season?

How are you paid for your work? In advance at the start of the season, or weekly/monthly per quota?

If you are paid in advance for your work, are you able to work that off by the end of the season? What happens if you can’t make enough bricks to work it off?

What do you think about the people that work with donkeys?

Would you like to own and work with donkeys?

Do you think owning a donkey has an impact on a person’s social status/their status within their community? In what ways?

Is owning a donkey something you have an interest in or aspire to? Why is that?

Would it have an impact on yours and your family’s life if you owned a donkey? In what way?

**Brick kiln owners/managers (non-equine owners)**

Can you tell me about where you and your family are from?

Who buys bricks from this kiln? What are they used for?

How important are donkeys to the running of this kiln? What is the contribution that they make?

What would happen at this kiln if there were no donkeys?

How do donkeys compare to having vehicles do the same work?

What do you think is the future for donkeys working in brick kilns?

Do any human development or environmental organisations work in this brick kiln, either currently or recently?

**Tekhidaars (contractors)**

Can you tell me about where you and your family are from?

How many of your family came here with you to the brick kiln?

How long have you worked in brick kilns?

Do all of the family members that came here with you work in the kiln too? What do they do?

How many donkeys do you own now?

How long have you worked with donkeys?

Can you tell us about your role as tekhidaar and what it involves? (how do you find people to come and work in the kilns? How do payments work between the brick kiln owner, yourself and the donkey owners, to get them to come and work in the kiln? Do you have any criteria about the numbers and characteristics of the donkeys that come to the kiln?)

Do you give advice to donkey owners about their animals?

Where do you go for advice or help with your donkeys?

Could you describe the types of shelter available for the equids? Why isn’t there any shelter?
