# Supplementary material for: Cultural “Blind Spots,” Social Influence and the Welfare of Working Donkeys in Brick Kilns in Northern India
Source: Front Vet Sci. 2020 Apr 29;7:214. doi: 10.3389/fvets.2020.00214 (PMC7201042; doi:10.3389/fvets.2020.00214)
Supplement: Supplementary file 2 [file Table_2.DOCX]

**Appendix 2. 2a. Body Condition Score Chart**

| **Condition Score** |  | **Neck and shoulders** |  | **Withers** |  | **Ribs and belly** |  |  | **Back and loins** |  | **Hindquarters** |
| --- | --- | --- | --- | --- | --- | --- | --- | --- | --- | --- | --- |
| 1.  POOR |  | Neck thin, all bones easily felt. Neck meets shoulder abruptly, shoulder bones felt easily, angular. |  | Dorsal spine of withers  Prominent and easily felt. |  | Ribs can be seen from a distance and felt with ease. Belly tucked up. |  |  | Backbone prominent, can feel dorsal and transverse processes easily. |  | Hip bones visible and felt easily (hock and pin bones). Little muscle cover. May be cavity under tail. |
| 2.  MODERATE |  | Some muscle development overlying bones. Slight step where neck meets shoulders. |  | Some cover over dorsal withers, spinous processes felt but not prominent. |  | Ribs not visible but can be felt with ease. |  |  | Dorsal and transverse processes felt with light pressure. Poor muscle development either side midline. |  | Poor muscle cover on hindquarters, hipbones felt with ease. |
| 3.  IDEAL |  | Good muscle development, bones felt under light cover of muscle/fat. Neck flows smoothly into shoulder, which is rounded. |  | Good cover of muscle/ fat over dorsal spinous processes withers flow smoothly into back. |  | Ribs just covered by light layer of fat/muscle, ribs can be felt with light pressure.  Belly firm with good muscle tone and flattish outline. |  |  | Cannot feel individual spinous or transverse processes.  Muscle development either side of midline is good. |  | Good muscle cover in hindquarters, hipbones rounded in appearance, can be felt with light pressure. |
| 4.  FAT |  | Neck thick, crest hard, shoulder covered in even fat layer. |  | Withers broad, bones felt with firm pressure. |  | Ribs dorsally only felt with firm pressure, ventral ribs may be felt more easily. Belly over developed. |  |  | Can only feel dorsal and transverse processes with firm pressure. Slight crease along midline. |  | Hindquarters rounded, bones felt only with firm pressure.  Fat deposits evenly placed. |
| 5.  OBESE |  | Neck thick, crest bulging with fat and may fall to one side.  Shoulder rounded and bulging with fat. |  | Withers broad, unable to feel bones |  | Large, often uneven fat deposits covering dorsal and possibly ventral aspect of ribs. Ribs not palpable.  Belly pendulous in depth and width. |  |  | Back broad, unable to feel spinous or transverse processes.  Deep crease along midline bulging fat either side. |  | Cannot feel hipbones, fat may overhang either side of tail head, fat often uneven and bulging. |


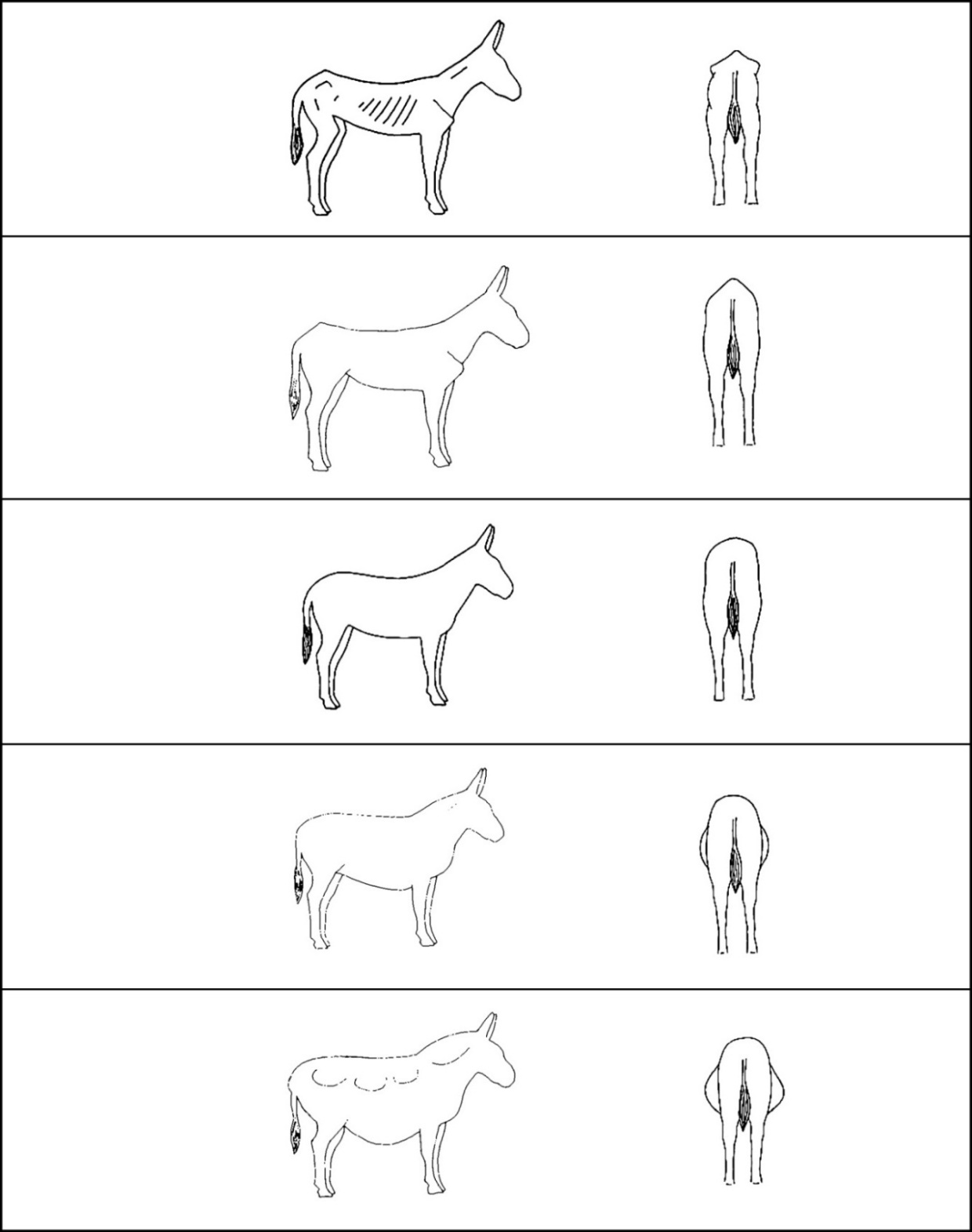


1. **Poor**
2. **Moderate**
3. **Ideal**

Fat deposits may be unevenly distributed especially over the neck and hindquarters. Some resistant fat deposits may be retained in the event of weight loss and/or may calcify (harden).

Careful assessment of all areas should be made and combined to give an overall score.

1. **Fat**
2. **Obese**

**2b. General Health**

INDICATOR 12 - HEALTH STATUS

| CATEGORY 12A - General health status  QUESTION  12-1 | Please indicate obvious signs of illness | 1 - No signs present  2 - Nasal discharge  3 - Eye discharge  4 - Signs of diarrhoea  5 - Unhealthy coat  6 - Significant discharge from the penis or vulva  7 - Abdominal pain  8 - Other (please specify) |
| --- | --- | --- |

NOTES select all the options that apply.

Question Choices:

| QUESTION 12-1 | 1 - No signs present | | |
| --- | --- | --- | --- |
| QUESTION 12-1 | | 2 - Nasal discharge | There is discharge, of any type, from 1 or both nares. |
| QUESTION 12-1 | | 3 - Eye discharge | There is discharge, of any type, from 1 or both eyes. |
| QUESTION 12-1 | | 4 - Signs of diarrhoea | The equid may pass diarrhoea during the examination or there may be evidence of faecal staining of the hindquarters and limbs. It is rare for donkeys to pass typical diarrhoea and faecal staining may be the only evidence of loose faeces. |
| QUESTION 12-1 | | 5 - Unhealthy coat | There are 1 or more signs of an unhealthy coat, e.g. dullness, alopecia, broken hairs, excessive scale |

| QUESTION 12-1 | 6 - Significant discharge from the penis or vulva | There is discharge present which cannot be attributed to normal activity. |
| --- | --- | --- |
| QUESTION 12-1 | 7 - Abdominal pain | Abdominal pain may be evident by any or all of; poor demeanour, flank watching, bruxism (teeth grinding), kicking at the abdomen, rolling, tenesmus (straining). Signs of many types of pain, including abdominal pain may be best observed before an equid is caught for examination. If this is the case, such signs, e.g. rolling will normally have been noticed at the start of the examination. Please note that donkeys may only demonstrate subtle signs of pain; dullness or a reported change in behaviour may be the only apparent symptom. Record any other signs observed that are not included in the list using the free text screen. Note that body condition has been assessed elsewhere (indicator 9). |
| QUESTION 12-1 | 8 - Other (please specify) | Record any other illness' using the free text screen. |
| QUESTION 12-2 | Please indicate apparent general health status of the equid? | 1 - Good  2 – Fair  3 - Poor |

NOTES

Look at the overall status of the equid; consider body condition, demeanour and external indicators of illness to rate the general health. Note that both underweight and overweight equids are at risk of poorer health.

| CATEGORY 12B - Coat  QUESTION 12-3 | Is the equid's coat healthy? | 1 - Healthy  2 - Unhealthy |
| --- | --- | --- |
|  |  |  |

NOTES

If the equid’s coat is dull, alopecic (patchy), broken haired or contains excessive scurf select option 2. Such signs may be an indication of poor skin or systemic health.
